# Prehospital stroke care in low- and middle-income countries: A World Stroke Organization (WSO) scientific statement

**DOI:** 10.1177/17474930251351867

**Published:** 2025-06-11

**Authors:** Jacqueline Bosch, Radhika Lotlikar, Rita Melifonwu, Tamer Roushdy, Ivy Sebastian, Siju V Abraham, Laura Benjamin, Dou Li, Gary Ford, Mirjam Heldner, Peter Langhorne, Renyu Liu, Emmie Malewezi, Olubukola A Olaleye, Jeyaraj Pandian, Gerard Urimubenshi, David Waters, Jing Zhao, Anthony Rudd

**Affiliations:** 1 IAHS School of Rehabilitation Science, McMaster University, Hamilton, ON, Canada; 2 Population Health Research Institute, McMaster University, Hamilton, ON, Canada; 3 Department of Neurology, Deenanath Mangeshkar Hospital and Research Center, Pune, Maharashtra, India; 4 Stroke Action Nigeria, Abuja, Nigeria; 5 Neurology Department, Faculty of Medicine, Ain Shams University, Cairo, Egypt; 6 Neurology Department, Christian Medical College and Hospital, Ludhiana, India; 7 Department of Emergency Medicine, Jubilee Mission Medical College and Research Institute, Thrissur, India; 8 Department of Neuroinflammation, Institute of Neurology, University College London, London, UL; 9 Beijing Emergency Medical Center, Beijing, China; 10 Division of Medical Sciences, University of Oxford and Oxford University Hospitals NHS Foundation Trust, Oxford, UK; 11 Stroke Center and Department of Neurology, University Hospital and University of Bern, Bern, Switzerland; 12 School of Cardiovascular & Metabolic Health, University of Glasgow, Glasgow, UK; 13 Departments of Anesthesiology and Critical Care, and Neurology, Perelman School of Medicine, University of Pennsylvania, Philadelphia, PA, USA; 14 Faculty of Health, Social Care and Medicine, Edge Hill University, Ormskirk, UK; 15 College of Medicine, University of Ibadan, Ibadan, Nigeria; 16 Neurology, Christian Medical College (CMC), Ludhiana, Punjab; 17 Department of Physiotherapy, College of Medicine and Health Sciences, University of Rwanda, Kigali, Rwanda; 18 Council of Ambulance Authorities, Hilton, SA, Australia; 19 Department of Neurology, Minhang Hospital, Fudan University, Shanghai, China; 20 King’s College London, London, UK

**Keywords:** Prehospital stroke care, low-and-middle-income countries, stroke action awareness, stroke education

## Abstract

Evidence-based prehospital stroke care is effective in reducing stroke-related mortality and morbidity. The crucial period from symptom awareness to presentation at the hospital, the first step in the World Stroke Organization Road Map to Quality Care, is under-resourced in the majority of low- and middle-income countries (LMICs). Key challenges focus on a lack of stroke action awareness as well as human resources trained in stroke care. We aimed to identify prehospital stroke practices in LMICs and identify where innovation may address service gaps. We conducted scoping reviews focused on key domains of prehospital stroke care in LMICs that include organization of services, stroke action awareness in the community, educating primary care physicians and traditional/faith healers, diagnostic tools for prehospital stroke detection, and emergency medical service (EMS) provision. We sought to determine current practices and gaps in LMICs and evidence on effective interventions to address gaps in each domain. Recommendations are provided identifying priority considerations in each domain, based on evidence, and where lacking, expert opinion. Key recommendations include the need for adequately funded national-level strategies for prehospital stroke care; stroke action awareness education for the public, primary care physicians, community health workers, EMSs, and traditional and faith healers; affordable imaging solutions; and approaches to create or improve prehospital EMS (e.g., protocols). We found that efforts, although few, have been made to address gaps in LMICs; however, they have rarely been evaluated, and it is unclear if they are sustained. The required elements necessary to improve prehospital services and stroke outcomes are known. Creativity is and perseverance are required for implementation to ensure sustainability. This scientific statement has been reviewed and approved by the World Stroke Organization Executive.

## Graphical abstract

**Graphical abstract:**
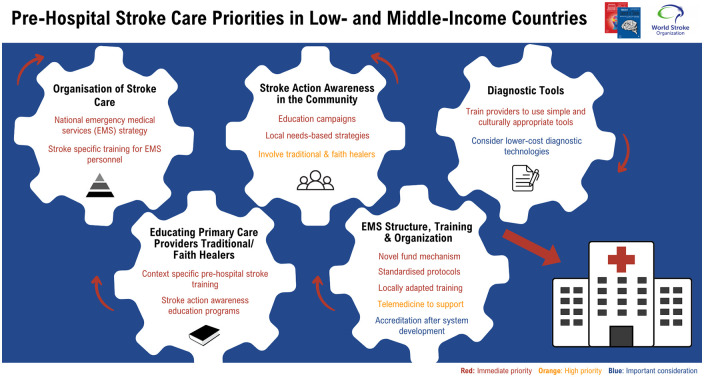

## Introduction

About 40% of morbidity and 50% of deaths in low- and middle-income countries (LMICs) result from conditions treatable with prehospital and emergency care.^1^ Stroke outcomes are directly dependent on rapid transport to hospital with highly trained personal,^2^ yet most of the world’s population does not have access to effective prehospital care because of a lack of services. The lack of service is most apparent in LMICs, where stroke burden is highest.^3^ A 2023 survey revealed that prehospital stroke care, which includes stroke action awareness by the public and health professionals, from the time of stroke symptom onset to arrival at a secondary care facility, is significantly more challenging in LMICs compared to high-income countries (HICs).^4^ A lack of human resources with specialty training in stroke care further limits the ability to address the lack of stroke action awareness and education.

Reducing global stroke burden requires immediate and significant improvement in prevention, stroke action awareness, and acute and post-acute treatment. This article, part of a World Stroke Organization (WSO) series, reviews prehospital stroke care in LMICs, highlights challenges, and offers recommendations for system and policies to improve stroke prehospital care and ultimately outcomes for stroke patients.

## Methods

### Writing group

Experts in stroke care and prehospital services in LMICs were invited to contribute based on their clinical or research expertise in prehospital care, with a focus on WSO Future Leaders. The group included stroke and emergency medicine physicians, paramedics, nurses, and rehabilitation specialists. Domains were identified, and smaller groups worked on specific sections, with regular meetings to ensure consistency.

### Topic content

A list of domains was created from the most recent guidelines and reviews on prehospital stroke care,^5–7^ with revision from the writing group to increase applicability to LMICs. In each section, we provide an overview of the current situation in LMIC settings, evidence from ongoing programs, and examples of successful initiatives and specific recommendations. Recommendations for each domain were created by the group and approved by all authors.

### Definitions

The World Bank classification was used to define LMICs.^8^ Low-resource settings were defined as those characterized by limited or no stroke units, revascularization treatments, trained stroke specialists, public health campaigns, emergency ambulances, and brain imaging technology. A country with a few high-resource centers but overall limited stroke care was still classified as a low-resource setting.

Priority ratings for each recommendation were defined as follows:

Immediate priority: sufficient evidence and considered important by the writing committee.
High priority: lack of evidence, yet considered important by the writing committee.
Important consideration: no evidence yet considered important by the writing committee.

### Literature review and methodology

It was recognized at the outset that publications on the topics specific to low-resource settings would be limited and, where available, may not be of high quality. As a result, we conducted a scoping review that included clinical trials, surveys, and qualitative reports. Databases searched included DORIS, EMBASE, MEDLINE, PUBMED, and SCOPUS, and searches were performed between February 2024 and February 2025. Search terms used were reflective of the specific domains. For example, for the domain “enhancing stroke awareness in the public,” terms such as public education, awareness, and stroke action awareness were combined with “low- and middle-income” and “developing countries.” Bibliographies were hand-searched. Preference was given to studies conducted in the last 10 years, unless more recent information was lacking. Study quality was not an inclusion criterion, as the purpose of this scoping review was descriptive. Point estimates of effect, where reported, were considered of limited value and likely to be highly unreliable. Further details on methodology can be found in the Supplementary Appendix.

## Current evidence

### Organization of stroke care

Integrated approaches to stroke care delivery are considered an effective way to reduce morbidity and mortality from stroke; however, their availability varies considerably across geographic regions.^9^ Although there are many challenges in setting up stroke services in LMICs, systematic reviews,^10,11^ reviews,^12^ consensus documents,^9,13^ and a World Stroke Organization–Lancet commission^14^ identified several reports on implementable and largely evidence-based prehospital stroke services in LMICs, many of which do not require major financial investment but do require commitment by local and central governments to deliver them effectively. Examples of these services are provided in Table 1.

**Table 1.** Examples of feasible evidence-based prehospital stroke services.

Culturally appropriate public health campaigns
Training of ambulance crews to identify stroke signs
Efficient models of ambulance transport and prenotification that identify stroke as a high priority
Stroke training programs for all levels of healthcare providers
Protocols for rapid evaluation and diagnosis and to guide acute care of stroke patients
Access to basic diagnostic services
Triaging eligible patients into thrombolysis-ready centers
Access to physicians with stroke expertise (may not always be stroke specialists)

### Recommendations

1. All states should have a funded strategy to support the development of EMS for patients with stroke. [Immediate Priority]
2. Health systems (national, state, and local) should develop an emergency medical system staffed by people who have received training in the recognition and immediate management of patients with possible stroke. [Immediate priority]

### Enhancing stroke action awareness and immediate action in the community

Stroke action awareness refers to the understanding of stroke risk factors, the recognition of stroke symptoms, and the need to seek immediate medical care when they occur.^15^ Lack of stroke action awareness in LMICs results in delays in prehospital and in-hospital management of acute stroke patients.^16,17^ These delays are at least partly due to limited health literacy and insufficient knowledge about stroke by the community, which contributes to poor health-seeking behaviors and may partly explain the differences in stroke care between HICs and LMICs.^12,18,19^ Financial constraints, as well as cultural and religious beliefs, also delay emergency medical care-seeking behaviors.^20^ For example, in Ghana, 25% believed their stroke was a spiritually related disease and, therefore, were more likely to seek spiritual help rather than attend a hospital.^21^

Predictors of greater stroke action awareness in LMICs include younger age, higher income, higher education, personal or family history of cardiovascular risk factors, stroke, or heart disease, and likely vary significantly between countries and urban and rural populations.^18,22^

Public education campaigns have shown an effect on improving stroke action awareness. A 2022 systematic review of public education campaigns identified 13 studies conducted in HICs.^23^ Each study used campaigns to educate on signs and symptoms, with four studies explicitly using the “Face-Arms-Speech-Time” (FAST) approach. There was a 20% improvement in symptom recognition and a 19% improvement in intention to use emergency services. The FAST Heroes Program was a study that educated children about FAST in school and encouraged them to share the information with their families at home. The study was conducted in 4 HICs and 1 MIC and enrolled 4200 parents and children from 11 countries, two of which were LMICs, and found that stroke knowledge increased for both children and parents.^24^ It was noted that this program is particularly suited for the multigenerational living arrangement often present in LMICs. Implementation of the language and culturally adapted Stroke 1-2-0 campaign in Shanghai, China (2016 and 2019), was also effective in increasing stroke knowledge.^25^ Stroke patients were eight times more likely to arrive at the hospital within 3 h of symptom onset and nine times more likely to use an ambulance after the campaign. In 2022, the Nepal Stroke Association and partners conducted a 6-month social media–based public stroke awareness campaign demonstrating the feasibility of using social media, with over 250,000 users accessing posts, although effectiveness was not assessed.^26^ It has also been suggested that Nepalese community health volunteers could play an important role in stroke action awareness, raising the possibility of involving the community health worker system in stroke symptom education campaigns.^27^ Many countries, HIC and LMIC, use the annual World Stroke Day to launch campaigns addressing various aspects of stroke action awareness. Key lessons from all these initiatives are that public education campaigns need to be sensitive to culture and language and recognize the role traditional healers and faith leaders may play in stroke action awareness.^15,28^

Transient ischemic attacks (TIAs) and minor strokes present a particular challenge in LMICs, as the public is often unaware of TIA symptoms and fails to seek help, or when they do, general practitioners, traditional healers, or paramedics may not recognize the symptoms.^29–32^ A study from North-west India reported a much lower incidence of TIA compared to HICs, which was attributed to poor awareness of stroke and TIA symptoms and limited access to resources.^33^

The Act FAST campaign had a positive effect on patient recognition of TIA or minor stroke in the United Kingdom and Australia, but was not successful in Qatar, potentially because of the differences in the populations.^34^

### Recommendations

1. Public education campaigns to increase stroke action awareness are necessary to improve stroke outcomes in low-resource settings and should recognize the predictors of stroke literacy and design campaigns that are sensitive to language and culture. [Immediate priority]
2. Local needs-based, simple strategies should be implemented to raise public awareness and action about the warning signs of TIA and encourage the use of prehospital EMS and rapid emergency department management. [Immediate priority]
3. Campaigns should consider the influence that traditional healers and faith leaders have in the community and how this could be used to improve stroke action awareness. [High priority]

### Educating primary care providers and traditional healers

Prehospital stroke care in LMICs is often provided by primary care providers, traditional healers, and, where they exist, paramedical staff.^35^ Insufficient knowledge and training among primary healthcare providers, coupled with a lack of standard prehospital clinical guidelines, results in substandard care, treatment delays, and poor outcomes.^36–38^ A study in Sri Lanka found that only 50% of primary care providers identified hypertension as the most important modifiable stroke risk factor, and only 13% could accurately define stroke. Even fewer were aware of the timing for thrombolysis.^39^ A study in Pakistan highlighted that 80% of community practitioners did not consider stroke survivors to require further referral, with three-quarters unaware of protocols and guidelines for thrombolysis and oral anticoagulation, and 74% believing that complete stroke recovery was impossible.^40^ Compromised quality care is estimated to contribute to between 5.7 and 8.4 million deaths annually in LMICs,^41^ one-third of which are related to cardiovascular disease, including stroke.^42^

Approaches used to improve acute in-hospital stroke care may be used to inform prehospital stroke care. In Nepal, a four-tiered approach, including training and quality monitoring, resulted in the implementation of key aspects of acute in-hospital stroke care in nine tertiary care hospitals by non-neurologists.^43^ This same training may be effective for primary care physicians, although it must be context-specific. The “Saving the Brain” initiative in East Delhi, India, used a hub-and-spoke approach to improve stroke knowledge among primary care physicians and was effective at improving outcomes.^44^ Nurses, doctors, and therapists without specialized neurological training were supported to create a stroke unit through remote training methods. Statistically significant improvements were seen for both process and clinical outcomes.^45^ A train-the-trainer approach was employed in Ghana for the prevention and treatment of stroke, and a similar approach could be used for prehospital care training.^46^ In Nigeria, one day of intensive stroke training for non-neurologists (primarily nurses and non-neurologist physicians) improved knowledge in all areas of stroke care, including stroke recognition.^47^ Models like the outpatient French SOS-TIA and British EXPRESS clinics have successfully reduced 90-day stroke recurrence by 80% through urgent evaluation of TIA.^48^ Key elements of prehospital stroke care may be improved using standardized tools, such as the National Institute of Health Stroke Scale (NIHSS). This has been used successfully by non-neurologists in high-resource settings and could result in more rapid assessment and improved communication between healthcare professionals, and may be particularly useful where primary healthcare assumes the role of emergency medical services (EMSs).^49–51^ Simplified approaches to differentiate atypical stroke symptoms like Triage-TiTRATE (Timing, Triggers and Targeted Examination for dizziness) or Explicit Diagnostic Criteria for TIA (EDCT) can help reduce the diagnostic errors in TIA diagnosis.^52–54^

Traditional healers are part of health practice in many settings, particularly where there is limited or no access to health care. Recently, there has been more discussion on the value of traditional and modern medicine working together in various settings (where it is not contra-indicated), particularly where there is limited or no access to health care.^55–57^ This approach holds promise for prehospital stroke care but requires time and commitment for relationship building.^58^

### Recommendations

1. Those involved in prehospital care must receive training on stroke diagnosis and optimal prehospital response. The method of training needs to be context-specific and should consider the components of previous campaigns that have been effective in LMICs. [Immediate priority]
2. Educational programs for general practitioners and paramedical staff should include TIA as well as stroke. [Immediate priority]
3. Traditional and faith healer training should also be considered. [Immediate priority]

### Diagnostic tools for prehospital stroke detection

The disparity in stroke outcomes between LMICs and HICs has been partly attributed to differences in access to diagnostic tools, capabilities, and resources.^11,59^

#### Technology for detecting stroke

The limitations in current practices for early stroke diagnosis have led to research in the use of diagnostic technologies in the prehospital setting.^60^ While portable computed tomography (CT) and magnetic resonance imaging (MRI) devices are under development, their high cost limits feasibility in LMICs. However, emerging point-of-care diagnostics, such as glial fibrillary acidic protein (GFAP)-based rapid tests for intracerebral hemorrhage detection, or near-infrared spectroscopy (NIRS) to rapidly differentiate ICH non-invasively in the periphery for rapid triaging and transport at the prehospital level, offer a promising, cost-effective solution.^28^ Developing artificial intelligence (AI)-driven algorithms could help to enhance stroke recognition or detection and trigger emergency medical dispatch via an immediate emergency call.

#### Clinical stroke detection tools

In many LMICs, tools aimed at identifying patients for thrombolysis or thrombectomy are irrelevant, but identifying stroke and getting them to the hospital for high-quality basic medical care is important. Instruments such as the Cincinnati Prehospital Stroke Scale (CPSS) are widely implemented globally due to their sensitivity, simplicity, and ease of use, making them accessible to both trained healthcare professionals and laypersons, an important advantage in regions with limited medical infrastructure.^20,61–63^ The Questionnaire to Verify Stroke Free Status (QVSFS), a community-level tool, was tested in Pakistan and showed good sensitivity (77.1%) and specificity (85.5%) compared to neurologists’ diagnosis.^64^ Integrating clinical tools into community health programs may improve stroke recognition and accelerate emergency response, even in the absence of neurologists and advanced diagnostic facilities.

### Recommendations

1. Clinician training and use of tools that are simple to learn and implement should be standard practice. These tools may need to be adapted for language and culture. [Immediate priority]
2. Lower-cost technology for stroke recognition may be beneficial for LMICs, including alternatives to MRI and CT for diagnosing stroke, such as smart wearables, AI, and biomarkers, but requires further development and evaluation before being implemented. [Important consideration]

### EMS Provision

EMS plays a critical role in minimizing time to treatment for stroke patients, yet significant barriers hinder its effectiveness in LMICs.

#### Structural and organizational barriers

EMS infrastructure in LMICs is frequently underdeveloped, lacking organized ambulance networks.^10,20,65^ Most patients have to rely on informal transportation to hospital, such as taxis or private cars, with the cost adding an additional barrier.^66–70^ Where ambulances are available, poor and congested roads delay access to timely treatment.^71^

EMS availability is better in urban areas, where hospitals are more readily accessible and better equipped.^72^ In rural areas, the response times are longer, EMS is scarcer, and patients often travel long distances to reach healthcare facilities.^35,69^ These factors result in delayed or inadequate care, with higher mortality rates among rural stroke patients.

Expanding access to ambulances through government subsidies or low-cost alternatives is essential. Successful models of layperson-operated ambulance services could be scaled in LMICs to reduce delays in reaching hospitals.^11,20^ In India, private–public initiatives to improve stroke care have been developed. The Angels Initiative is working with EMRI Green Health Services, a GVK Enterprise, to train over 9000 emergency medical response (EMR) teams and to improve prehospital care across India.^73^

In densely populated cities with poor traffic infrastructure, strengthening public educational campaigns and implementing telemedicine may be necessary to support timely stroke care. Telemedicine offers an innovative solution by facilitating triage, addressing the shortage of skilled personnel, and expanding access to care, including in rural areas. Although evidence from HICs is promising, the effectiveness of telemedicine in LMIC remains uncertain.^74^

Establishing cost-effective stroke units that connect prehospital care with hyperacute hospital care may also improve long-term outcomes. For example, in Colombia, prehospital mobile phone notification helped coordinate referral efforts at the receiving stroke unit, reduced door-to-needle times, and consequently improved stroke outcomes.^75^

#### Training EMS staff

An effective EMS system includes well-trained, skilled prehospital and in-hospital EMS personnel who provide rapid response, stroke identification, transfer, and prenotification.^10,14^ Most LMICs have few trained EMS personnel despite clear guideline recommendations for EMS personnel training, triage protocols, and rapid identification of stroke.^6^ An international survey of eight African countries demonstrated that only 20% offered training to call handlers or paramedics.^4,76^ A Ghanaian study of major referral hospitals found that only 9.1% had local protocols for prehospital care.^77^ Locally applicable standard operating procedures can also improve stroke care in LMICs.^78^

EMS education strategies need to take into consideration local needs, resources, and available services.^4,76,79^ In Nepal, a scenario-based training protocol for dispatchers (call handling) has been developed and is now implemented in a large ambulance dispatch network.^80^ As previously noted, simple prehospital screening and assessment tools (e.g., Face-Arm-Speech-Time (FAST), Rapid Arterial oCclusion Evaluation (RACE), G-FAST (Gaze-Face-Arm-Speech-Time), CG-FAST (Conveniently Grasped Field Assessment Stroke Triage)) can be used by paramedics to identify ischemic stroke but require training.^63^

Open-source training opportunities, such as the ASLS: Angels Advanced Stroke Life Support for Prehospital Providers provide basic training.^81^ The previously mentioned “Save the Brain” initiative in East Delhi provided training for paramedics.^44^ In 2016, the Iranian Ministry of Health implemented the “Stroke Program 724” to improve stroke outcomes in Iran; this plan included training of emergency staff involved in prehospital treatment.^82^ These training approaches seem to be low-cost and effective, making them particularly suited to implementation in LMICs

#### Accreditation: A tool for quality improvement of EMS services

Accreditation is a voluntary program where trained external reviewers evaluate a healthcare organization’s compliance with pre-established performance standards to promote continuous improvement and optimal quality.^83^ The Joint Commission offers a Primary Stroke Center (PSC) Certification; however, it does not certify the prehospital components.^84^ In 2022, the World Stroke Organization launched a free Certification of Stroke Centers; however, EMS is not included.^85^ There are efforts aimed at supporting quality improvement in EMS services. The Registry of Stroke Quality Care (RES-Q) provides support for quality monitoring of EMS service provision and provides the EMS Angels Awards to those centers meeting EMS service delivery criteria.^86^ Evidence supporting the use of accreditation to improve prehospital stroke care is growing, although findings remain mixed and are primarily from HICs.^87,88^

### Recommendations

#### Structural and organizational barriers

1. Expanding access to ambulances through government subsidies or low-cost alternatives is essential. Successful models of layperson-operated ambulance services should be scaled in LMICs to reduce delays in reaching hospitals. [Immediate priority]
2. Public and private partnerships should be considered to develop prehospital ambulance systems. [Immediate priority]
3. Standardized protocols developed through government and non-governmental organization (NGO) collaboration are crucial for uniform stroke care, although implementation remains challenging even in HIC. [Immediate priority]
4. Telemedicine should be considered as an innovative solution that supports triaging, addresses the shortage of skilled personnel, and expands access, ultimately ensuring timely interventions. [High priority]

#### Training

1. Training initiatives should be adapted to meet local needs; consider scenario-based training. [Immediate priority]

#### Accreditation

1. In low-resource settings, where evidence for EMS accreditation is limited, prioritizing it may not be the most effective use of resources; instead, efforts should focus on strengthening EMS infrastructure, workforce training, and standardized protocols, developed through government and NGO collaboration, to improve prehospital stroke care. [Important consideration]

### Limitations

While care was taken to conduct a comprehensive search of the literature, some initiatives may have been missed. It is hoped this review will inspire further efforts to compile all available evidence. The writing group represents many professions involved in the prehospital stroke care, but could not represent all relevant specialties from all geographical areas. The perspective of stroke survivors and public health policy experts was not part of this review.

## Conclusion

Primary prevention is the key to any public health endeavor, but when that fails, prehospital care is often the first point of contact. This paper reviews the current state of prehospital stroke care in LMICs and highlights some of the efforts that have been undertaken to address the gaps. Stroke research agendas in LMICs must now move beyond describing the problems to testing and implementing effective solutions. International and national governmental and stroke organizations can lead public and professional educational campaigns advocating for improved prehospital stroke care. Contextually relevant guidelines could help to establish common care pathways, and health care professionals can try and implement better services within their environment. System-level initiatives, with embedded outcome evaluation and quality improvement practices, are required immediately for change to be substantive and sustainable. Although there is a need for immediate action at every level in the stroke care pathway in LMICs, the prehospital/hospital entry phase of stroke care in LMICs should be an urgent and paramount priority. Based on the recommendations provided, we have summarized below the most urgent research and policy priorities:

1. Better epidemiologic evidence on the use of prehospital care in LMICs will enable gap analysis and cost-effectiveness studies.
2. Identifying effective ways to increase stroke action awareness in different cultural settings.
3. Evaluation of low-cost alternatives to paramedic- staffed emergency services (e.g. rapid response teams).
4. Evaluation of telemedicine/mobile apps and AI in LMIC to diagnose and deliver emergency care in the prehospital setting.
5. Evaluation of protocol-driven care in low-resource settings.
6. Evaluation of low-cost low imaging technology for urgent stroke or TIA assessment and management in low-resource settings.
7. The development of national prehospital stroke policy based on existing evidence, even if not from LMICs or directly from stroke, with a concurrent research agenda to determine effectiveness. The policy needs to be based on prehospital indicators that meet local goals.

## Supplemental Material

sj-docx-1-wso-10.1177_17474930251351867 – Supplemental material for Prehospital stroke care in low- and middle-income countries: A World Stroke Organization (WSO) scientific statement Supplemental material, sj-docx-1-wso-10.1177_17474930251351867 for Prehospital stroke care in low- and middle-income countries: A World Stroke Organization (WSO) scientific statement by Jacqueline Bosch, Radhika Lotlikar, Rita Melifonwu, Tamer Roushdy, Ivy Sebastian, Siju V Abraham, Laura Benjamin, Dou Li, Gary Ford, Mirjam Heldner, Peter Langhorne, Renyu Liu, Emmie Malewezi, Olubukola A Olaleye, Jeyaraj Pandian, Gerard Urimubenshi, David Waters, Jing Zhao and Anthony Rudd in International Journal of Stroke
