# Supplementary material for: Prehospital stroke care in low- and middle-income countries: A World Stroke Organization (WSO) scientific statement
Source: Int J Stroke. 2025 Jun 11;20(8):918–27. doi: 10.1177/17474930251351867 (PMC12446713; doi:10.1177/17474930251351867)
Supplement: sj-docx-1-wso-10.1177_17474930251351867 – Supplemental material for Prehospital stroke care in low- and middle-income countries: A World Stroke Organization (WSO) scientific statement [file sj-docx-1-wso-10.1177_17474930251351867.docx]

**Supplemental Appendix – Key Elements of Scoping Review Methodology to Guide Work in Each Domain**

**Objective of the Paper**

The review will present the research literature, summarise the data about service provision where available and suggest areas for research and suggestions for approaches to quality improvement.

For each section there should be:

1. A review of the literature (see below for inclusion criteria)
2. Recommendations for research in areas of uncertainty
3. Recommendations for policy at international (WSO, WHO etc.), national governmental policy, local policy and clinical practice.
4. Case studies illustrating good practice.

**Searching for Evidence**

The search for evidence should focus on seeking good previous reviews and high-quality basic research/reports.

While the focus is on LMIC, papers from HIC can be used to inform the discussion/conclusions. However, the focus should be on strategies that have been attempted or have worked in LMIC.

*Definition of low resource settings:*

- No organized stroke unit/stroke centre or hospitals
- Lack of time dependent pharmacological (tPA) or interventional capabilities (endovascular treatment)
- No specialized trained health professionals  e.g. neurologists specializing in stroke care
- Lack of a public health campaign for triaging stroke
- Limited access to technology e.g., urgent brain imaging
- Limited or no access to ambulances for medical emergencies
- Having a minority of high resource sites in an otherwise low resource settings does not preclude the consideration of that country region as “low resource:
- We are using 2024 World Bank criteria for defining low- and middle-income countries

*Databases to consider*

DORIS (Database of Research in Stroke, <https://www.askdoris.org/openversion/main.asp?opt=2709>)

EMBASE

Google Scholar

SCOPUS

MEDLINE

PUBmed

However also consider which local journals may be applicable and not indexed.

NOTE:

- If an article is identified that you are unable to access please contact Jackie
- We could not get a COVIDENCE license for use by the group
- If you do not have access to a specific database and would like to re-run your search, please contact Jackie

*Defining the domains*

The final domains are as follows:

Public education

Organization of stroke care

Clinical assessment tools for low resource countries

Non-neurologist doctor education including traditional healers

EMS Education

Provision of EMS in urban vs rural

Use of EMS accreditation as a tool for quality improvement

Technology and diagnostic tools for prehospital use and applicability

Apps for stroke recognition

Management of TIA, minor stroke in low resource settings + Provision of urgent stroke care in receiving hospitals

TIA identification

Each domain working group is asked to come up with a list of search terms to be used to identify literature within the specific domain. Once the reviews are completed some domains may be collapsed together, shortened or removed to comply with the 6,000-word limit of the journal.

An example of a search strategy and results is provided in Appendix A.

**Data Extraction**

Groups are asked to compile their study results, review the studies to determine those that meet the criteria for the domain, and determine which elements of the study are to be included. For example, there may be a study protocol describing a suggested approach to pre-hospital stroke in LMICs for which just the description of the intervention is included, or there may be a policy document that includes content on the specific domain aspect. Groups must come to consensus regarding content to be included before submitting their draft.

**Appendix A: STROKE PUBLIC EDUCATION IN LMICs**

**KEY WORDS:** public education, awareness, stroke action awareness, low and middle income countries, developing countries

| **No.** | **Database.** | **References** | **Location** |
| --- | --- | --- | --- |
|  | SCOPUS  PUBMED  DORIS | Reference Manager@ Refworks |  |
| 1 |  | Baby, P., Ravi, R. K., & Kathyayani, B. V. (2023). A community-based survey on stroke awareness in underprivileged urban areas of bangalore, india.*Africa Journal of Nursing & Midwifery, 25*(2) | Bangalore  India |
| 2 |  | Baatiema, L., Sanuade, O., Kuumuori Ganle, J., Sumah, A., Baatiema, L., & Sumankuuro, J. (2021). An ecological approach to understanding stroke experience and access to rehabilitation services in ghana: A cross‐sectional study.*Health & Social Care in the Community, 29*(5), e67–e78. | Ghana |
| 3 |  | Dalal, P., Bhattacharjee, M., Vairale, J., & Bhat, P. (2007). UN millennium development goals: Can we halt the stroke epidemic in india?*Annals of Indian Academy of Neurology, 10*(3), 130–136. | India |
| 4 |  | Eltayib, E. M., Jirjees, F., Suliman, D., AlObaidi, H., Ahmed, M., Kharaba, Z. J., . . . ALSalamat, H. (2024). Stroke awareness and knowledge in sudan: A cross-sectional analysis of public perceptions and understanding.*Frontiers in Public Health, 12*, 1362979. | sudan |
| 5 |  | Farrag, M. A., Oraby, M. I., Ghali, A. A., Ragab, O. A., Nasreldein, A., Shehata, G. A., . . . Abd-Allah, F. (2018). Public stroke knowledge, awareness, and response to acute stroke: Multi-center study from 4 egyptian governorates.*Journal of the Neurological Sciences, 384*, 46–49. | Egypt |
| 6 |  | Fitzpatrick, A. L., Steinman, L. E., Tu, S., Ly, K. A., Ton, T. G., Yip, M., & Sin, M. (2012). Using photovoice to understand cardiovascular health awareness in asian elders.*Health Promotion Practice, 13*(1), 48–54. | Asia |
| 7 |  | Halpin, H. A., Morales-Suárez-Varela, M. M., & Martin-Moreno, J. M. (2010). Chronic disease prevention and the new public health.*Public Health Reviews, 32*, 120–154. | LMIC / Developing country perspective |
| 8 |  | Hamdy, H., Abdel-Monem, A., Emara, T. H., Moustafa, R. R., Bar, A. A., Abuzeid, S. H., . . . El-Mously, S. (2013). Knowledge and attitudes towards stroke among workers in two university hospitals.*Egyptian Journal of Neurology, Psychiatry & Neurosurgery, 50*(1 | Egypt |
| 9 |  | He, F. J., Jenner, K. H., & MacGregor, G. A. (2010). WASH—world action on salt and health.*Kidney International, 78*(8), 745–753. | LMIC / Developing country |
| 10 |  | Heizhati, M., Li, N., Zhang, D., Abulikemu, S., Chang, G., Hong, J., . . . Duiyimuhan, G. (2021). Government‐Expert joint intervention with treatment algorithm and improved hypertension management and reduced stroke mortality in a Primary‐Care setting.*International Journal of Hypertension, 2021*(1), 9661576. | Emin, China |
| 11 |  | Kamran, S., Bener, A. B., Deleu, D., Khoja, W., Jumma, M., Al Shubali, A., . . . Al Khabouri, J. (2008). The level of awareness of stroke risk factors and symptoms in the gulf cooperation council countries: Gulf cooperation council stroke awareness study.*Neuroepidemiology, 29*(3-4), 235–242. | Arabian Gulf |
| 12 |  | Khan, F., Gaowgzeh, R. A. M., Saif, A. A., Chevidikunnan, M. F., Soman, A., Mazi, A., . . . Anjamparuthikal, H. (2021). Effect of community education program on stroke symptoms and treatment on school and college students from south india: A longitudinal observational study. Paper presented at the *Healthcare, , 9*(12) 1637. | India |
| 13 |  | Mat Said, Z., Tengku Ismail, T. A., Abdul Hamid, A., Sahathevan, R., Abdul Aziz, Z., & Musa, K. I. (2022). The malay version of the attitudes and beliefs about cardiovascular disease (ABCD-M) risk questionnaire: A translation, reliability and validation study.*BMC Public Health, 22*(1), 1412. | Malaysia |
| 14 |  | Mensah, G. A. (2003). A heart-healthy and “Stroke-free” world through policy development, systems change, and environmental supports.*Ethnicity & Disease, 13*, 4–12. | Sub-Sahara Africa |
| 15 |  | Nowrin, I., Bhattacharyya, D. S., & Saif-Ur-Rahman, K. M. (2022). Community-based interventions to prevent stroke in low-income and middle-income countries: A protocol for a systematic review and meta-analysis.*BMJ Open, 12*(8), e063181. | LMIC perspective |
| 16 |  | Ookeditse, O., Ookeditse, K. K., Motswakadikgwa, T. R., Masilo, G., Bogatsu, Y., Lekobe, B. C., . . . Johnsen, S. H. (2022). Public and outpatients’ awareness of calling emergency medical services immediately by acute stroke in an upper middle-income country: A cross-sectional questionnaire study in greater gaborone, botswana.*BMC Neurology, 22*(1), 347. | Garbone, Botswana |
| 17 |  | Osama, A., Ashour, Y., El-Razek, R. A., & Mostafa, I. (2019). Public knowledge of warning signs and risk factors of cerebro-vascular stroke in ismailia governorate, egypt.*The Egyptian Journal of Neurology, Psychiatry and Neurosurgery, 55*, 1–6. | Egypt |
| 18 |  | Pu, C., Guo, J., & Sankara, P. (2020). Comparison of knowledge on stroke for stroke patients and the general population in burkina faso: A cross-sectional study.*AIMS Public Health, 7*(4), 723. | Burkina Faso |
| 19 |  | Ramadan, J., Vuori, I., Lankenau, B., Schmid, T., & Pratt, M. (2010). Developing a national physical activity plan: The kuwait example.*Global Health Promotion, 17*(2), 52–57. | Kuwait |
| 20 |  | Saadatnia, M., Hajiannejad, N., Yazdabadi, A., Tajmirriahi, M., & Nasr, M. (2021). Public stroke knowledge, awareness, and response to acute stroke in isfahan iran: What is less or misinterpreted in developing countries.*Journal of Stroke and Cerebrovascular Diseases, 30*(6), 105670. | Isfahan Iran |
| 21 |  | Sese, L. V. C., & Guillermo, M. C. L. (2023). Strengthening stroke prevention and awareness in the philippines: A conceptual framework.*Frontiers in Neurology, 14*, 1258821. | Philipines |
| 22 |  | Silva, G. S., & Rocha, E. (2024). Developing systems of care for stroke in resource-limited settings. Paper presented at the *Seminars in Neurology,* | LMIC \ Developing country perspective |
| 23 |  | Skar, P., Young, L., & Gordon, C. (2015). Changes in blood pressure among users of lay health worker or volunteer operated community-based blood pressure programs over time: A systematic review protocol.*JBI Evidence Synthesis, 13*(10), 30–40. | LMIC perspective |
| 24 |  | Tunkl, C., Paudel, R., Thapa, L., Tunkl, P., Jalan, P., Chandra, A., . . . Bajaj, S. (2023). Are digital social media campaigns the key to raise stroke awareness in low-and middle-income countries? A study of feasibility and cost-effectiveness in nepal.*Plos One, 18*(9), e0291392. | Nepal |
| 25 |  | Walelgn, N., Abyu, G. Y., Seyoum, Y., Habtegiorgis, S. D., & Birhanu, M. Y. (2021). The survival status and predictors of mortality among stroke patients at north west ethiopia.*Risk Management and Healthcare Policy,*, 2983–2994. | Ethiopia |
